# Lithium during pregnancy and after delivery: a review

**DOI:** 10.1186/s40345-018-0135-7

**Published:** 2018-12-02

**Authors:** Eline M. P. Poels, Hilmar H. Bijma, Megan Galbally, Veerle Bergink

**Affiliations:** 1 Department of Psychiatry, Erasmus University Medical Centre Rotterdam, Rotterdam, The Netherlands; 2 Department of Obstetrics and Gynaecology, Division of Obstetrics and Prenatal Medicine, Erasmus University Medical Centre Rotterdam, Rotterdam, The Netherlands; 3 School of Psychology and Exercise Science, Murdoch University, Murdoch, Australia; 4 Department of Psychiatry and Department of Obstetrics, Gynecology and Reproductive Science, The Blavatnik Women’s Health Research Institute, Icahn School of Medicine at Mount Sinai, 1425 Madison Avenue, Room L4-34, New York City, NY 10029 USA

**Keywords:** Lithium, Pregnancy, Perinatal, Bipolar disorder, Postpartum psychosis, Congenital malformations, Review, Breastfeeding, Delivery, Neurodevelopment

## Abstract

Lithium is an effective treatment in pregnancy and postpartum for the prevention of relapse in bipolar disorder. However, lithium has also been associated with risks during pregnancy for both the mother and the unborn child. Recent large studies have confirmed the association between first trimester lithium exposure and an increased risk of congenital malformations. Importantly, the risk estimates from these studies are lower than previously reported. Tapering of lithium during the first trimester could be considered but should be weighed against the risks of relapse. There seems to be no association between lithium use and pregnancy or delivery related outcomes, but more research is needed to be more conclusive. When lithium is prescribed during pregnancy, lithium blood levels should be monitored more frequently than outside of pregnancy and preferably weekly in the third trimester. We recommend a high-resolution ultrasound with fetal anomaly scanning at 20 weeks. Ideally, delivery should take place in a specialised hospital where psychiatric and obstetric care for the mother is provided and neonatal evaluation and monitoring of the child can take place immediately after birth. When lithium is discontinued during pregnancy, lithium could be restarted immediately after delivery as strategy for relapse prevention postpartum. Given the very high risk of relapse in the postpartum period, a high target therapeutic lithium level is recommended. Most clinical guidelines discourage breastfeeding in women treated with lithium. It is highly important that clinicians inform and advise women about the risks and benefits of remaining on lithium in pregnancy, if possible preconceptionally. In this narrative review we provide an up-to-date overview of the literature on lithium use during pregnancy and after delivery leading to clinical recommendations.

## Background

Lithium therapy has a well-established evidence base as a long-term maintenance treatment for bipolar disorder with demonstrated efficacy in reducing both manic and depressive relapse and anti-suicidal properties (Geddes and Miklowitz 2013). Bipolar disorder often has its onset before the age of 25 years (Merikangas et al. 2011), and as such lithium is frequently prescribed to women of childbearing age. However, there is enormous global variance in prescription patterns of lithium and recommendations for its use during the perinatal period (defined as pregnancy and the first year postpartum). In general, data on the prevalence of lithium use during pregnancy are scarce with the exception of population-based studies from Denmark and the UK. In a recent clinical overview, the Danish author Larsen and colleagues recommended lithium as the first-line mood-stabilizing treatment during pregnancy (Larsen et al. 2015). Despite this recommendation, only 16% (53/336) of women with bipolar disorder redeemed at least one lithium prescription during pregnancy and only 6.3% of women used lithium in the third trimester, indicating that the majority of women discontinued lithium during pregnancy (Broeks et al. 2017). Similarly, in the UK discontinuation rates of lithium during pregnancy is high with a study of pregnant women showing that only 17 out of 52 pregnant women continued lithium use during pregnancy (McCrea et al. 2015). This pattern of discontinuation of lithium before pregnancy is supported by the NICE guideline where it is recommended to “not offer lithium to women who are planning a pregnancy or pregnant, unless antipsychotic medication has not been effective” (National Collaborating Centre for Mental H 2014). For most other countries information on lithium use during pregnancy is lacking. In a recent meta-analysis on bipolar disorder in the perinatal period, 5700 bipolar pregnancies (n = 37 studies) were included (Wesseloo et al. 2016). Of these, information on medication use was only available for 445 bipolar pregnancies (60 women with various medication including lithium and 385 women without medication). Most of these lithium users came from the Netherlands, which can be explained by the recommendation in the Dutch guidelines to list lithium as a first line treatment option during pregnancy (Trimbos-instituut 2015). In the Australian Clinical Practice Guideline on perinatal mental health, no specific recommendation was made to continue or discontinue lithium during pregnancy, but rather proposes care pathways for both situations (Austin et al. 2017). Altogether, guidelines give inconsistent and highly variable information regarding the safety of lithium use during pregnancy. A comprehensive Canadian review of recommendations for the treatment of bipolar disorder during pregnancy recommended: “Women at risk for new onset or relapse of a mood episode who are not on maintenance treatment should be considered for trial of a mood stabilizer other than valproate, or an atypical antipsychotic drug” (Sharma and Sharma 2016).

To guide clinicians in their decision making we provide a narrative review of the literature on efficacy of lithium use in the perinatal period and the risks for mother and child.

## Lithium during pregnancy

### Efficacy

To date the literature on the impact of pregnancy on the course of bipolar disorders is inconsistent. Previous studies suggested that women with bipolar disorder may have a lower risk of relapse during pregnancy, when compared to the period before or after (Grof et al. 2000). A recent systematic review concluded that based on the literature to date the question of how pregnancy affects the course of bipolar disorder can’t be answered (Salim et al. 2018). Viguera et al. showed that in women who discontinued mood stabilizing treatment including lithium during pregnancy (n = 62), the relapse risk was two times increased compared to women who continued treatment (n = 27) (Viguera et al. 2007). In the postpartum period there is a high risk of a bipolar episode and hospitalization for psychiatric morbidity (Munk-Olsen et al. 2006; Harlow et al. 2007; Di Florio et al. 2013). A perinatal history of affective psychosis or depression is the most important risk factor, as reported in a recent cohort study investigating risk factors for postpartum recurrence in bipolar disorder (Di Florio et al. 2018). Unfortunately, this study did not investigate the effect of medication use during pregnancy on the risk of recurrence. A recent meta-analysis showed significantly higher postpartum relapse rates in women without medication during pregnancy (N = 385; 66%, 95% CI 57–75) as compared to women using prophylactic medication (N = 60, 23%, 95% CI 14–37) (Wesseloo et al. 2016). Of these 60 patients with prophylactic medication during pregnancy, the majority used lithium (Bergink et al. 2012; Austin 1992; Freeman et al. 2002; Bilszta et al. 2010). Hence, lithium prophylaxis during pregnancy in women with bipolar disorder might be important not only to maintain mood stability during pregnancy, but also for postpartum relapse prevention.

Interestingly, a recent population based cohort study reported that lamotrigine during pregnancy was not inferior to lithium in the prevention of severe postpartum episodes (Wesseloo et al. 2017). However, the authors point out the likely influence of confounding by indication since lamotrigine was primarily prescribed to women with a vulnerability for depressive episodes, while lithium was primarily prescribed to women with a history of manic episodes. Therefore, this finding requires replication in studies that can account for diagnosis, variant and severity of illness.

### Dosing and monitoring of blood levels during pregnancy and around delivery

Lithium has a narrow therapeutic range of 0.5–1.2 mmol/L and higher levels may lead to toxicity (Oruch et al. 2014). Excretion of lithium is almost exclusively renal, hence blood plasma levels mainly depend on intravascular volume and glomerular filtration rate (GRF) (Oruch et al. 2014; Grandjean and Aubry 2009). As pregnancy progresses total body water, plasma volume and GFR are increased (Pariente et al. 2016) with GFR rising from as early as 6 weeks gestation up to 50% over non-pregnant women by the end of the first trimester (Davison 1984). Clinical studies have shown lithium blood levels to decrease significantly during pregnancy (Wesseloo et al. 2017; Westin et al. 2017). An average decrease of 24% in the first trimester, 36% in second trimester and 21% in third trimester was described. Creatinine blood levels showed a similar longitudinal pattern, showing that indeed changes in lithium blood level reflect changes in renal physiology.

In summary, first and second trimester are characterised by a significant decrease of lithium blood levels with a risk of subtherapeutic levels. In third trimester and the postpartum, lithium levels gradually return to their preconception level which implicates that in this period clinicians need to be aware of the risk of lithium intoxication. Close monitoring and dose adjustment is needed with conditions such as hyperemesis gravidarum, pre-eclampsia, impaired renal function, concomitant medication or acute blood loss occur, as these conditions increased the risk of toxicity (Handler 2009; Blake et al. 2008). Furthermore, as lithium levels in the fetus equal those in the mother, changes in dosing may impact fetal health and increase the risk of complications (Newport et al. 2005). A multiple day dosing regime has been proposed to minimise fetal risk by minimising peak lithium levels (Horton et al. 2012). However, multiple day dosing has been associated with an increased risk of renal side effects and as a consequence possible non-adherence (Singh et al. 2011). Therefore, twice daily dosing seems to be preferred to more frequent administration.

The above described dynamic changes in GFR and maternal haemodynamics during pregnancy necessitate monthly monitoring of lithium blood levels until 34 weeks and weekly monitoring thereafter until delivery (Wesseloo et al. 2017).

Several authors and guidelines have suggested to decrease or discontinue lithium treatment when in labour in order to minimise lithium side-effects in the neonate (National Collaborating Centre for Mental H 2014; Trimbos-instituut 2015; Newport et al. 2005). However, there is currently no evidence that suggests this strategy decreases the risk of perinatal and infant complications and this strategy has to be weighed against the risk of maternal relapse during a high-risk period. Both Deligiannidis et al. and Wesseloo et al. have recommended careful lithium blood level monitoring instead of discontinuation in all cases (Deligiannidis et al. 2014). Lithium blood levels should be measured before and 24 h after delivery and adequate fluid management is important to prevent dehydration. Lithium blood level, as well as thyroid-stimulating hormone (TSH) and free thyroxine (T4) should be evaluated in umbilical cord blood sample (Trimbos-instituut 2015). Nephrotoxic medication and nonsteroidal anti-inflammatory drugs should be avoided (Deligiannidis et al. 2014). When considering anaesthesia options during delivery, drug interactions with lithium should be taken into account. Lithium potentiates succinylcholine and pancuronium and can be expected to potentiate other depolarising and non-depolarizing muscle relaxants (Blake et al. 2008). Close monitoring of neuromuscular function is therefore required. Regional anaesthesia is considered to be safe (Blake et al. 2008).

### Obstetric complications

When investigating the effect of lithium exposure on obstetric complications in cohort studies it is important to consider that bipolar disorder, the indication for which lithium is often prescribed, is associated with obstetric complications independent of medication. In specific, women with bipolar disorder are at increased risk of antepartum hemorrhage, placental abnormalities and caesarean section (Boden et al. 2012; Jablensky et al. 2005). The mechanism underlying this increased risk for women with bipolar disorder is unclear but psychosocial stress accompanied by high cortisol levels, comorbidity and lifestyle factors might play a role (Boden et al. 2012). In a recent shared protocol meta-analysis of 727 lithium exposed pregnancies and 21,397 pregnancies in disease matched controls lithium use during pregnancy was not associated with preeclampsia, diabetes during pregnancy, fetal distress, postpartum hemorrhage or caesarean section (Munk-Olsen et al. 2018). Additionally, in two studies the rates of obstetric complications were not higher in women who continued lithium during pregnancy compared to women who discontinued lithium before or early in pregnancy (Petersen et al. 2016; Frayne et al. 2017). Table 1 presents an overview of the results from observational cohort studies on obstetric complications of lithium use during pregnancy. Results of these studies should be interpreted considering several methodological limitations, i.e. the sample size of two studies was very small and these studies did not correct for confounding variables and timing, duration or dose of the exposure.

**Table 1:** Obstetric outcome after lithium treatment during pregnancy: findings from clinical cohort studies

Study	Design	Sample size	Findings
Petersen et al. (2016)	Registry-based study	Exposed = 35 Disease matched non-exposed = 84 Controls = 320.853	No difference in the rate of caesarean sections
Frayne et al. (2017)	Cohort study	Exposed = 33	No difference in the rate of obstetric complications between the women that continued (n = 19) or discontinued (n = 14) lithium
Munk-Olsen et al. (2018)	Meta-analysis (six study sites)	Exposed = 727 Disease matched controls = 21,397	No association between lithium exposure in utero and preeclampsia (OR 0.97, 95% CI 0.52–1.80), gestational diabetes (OR 1.20, 95% CI 0.81–1.78), fetal distress (OR 1.00, 95% CI 0.76–1.32), postpartum hemorrhage (OR 1.28, 95% CI 0.64–2.57) and caesarean section (OR 0.94, 95% CI 0.66–1.33)

*OR* odds ratio, *CI* confidence interval

Polyhydramnios has not been investigated in observational cohort studies, but has been described in two case reports (Ang et al. 1990; Krause et al. 1990). This warrants further investigation because polyuria is a well-known side effect of lithium and fetal polyuria could lead to polyhydramnios. In summary, while women with bipolar disorder have an increased risk of obstetric complications, there seems no association between lithium use during pregnancy and pregnancy or delivery related outcomes.

## Consequences for the developing child

Lithium freely crosses the placental barrier and lithium concentrations equilibrate between maternal and fetal circulation (Newport et al. 2005). Hence maternal lithium therapy results in fetal lithium exposure. We provide a summary of published results from investigations on the short- and long-term consequences of intrauterine exposure to lithium.

### Congenital malformations

The first trimester of pregnancy is crucial to the normal development of the fetus. Since in this period all major body organs are forming, the fetus is susceptible to damage from teratogens and this has raised some concerns about the possible teratogenicity of lithium use during the first trimester. In this review we summarise the results from clinical cohort studies investigating the risk of congenital malformations after lithium use during pregnancy, an overview of these studies is presented in Table 2.

**Table 2:** Findings from clinical cohort investigations on the association between in utero exposure to lithium and congenital malformations

Study	Design	Sample size	Findings
Schou et al. (1973)	Cohort study	Exposed = 118	Nine children with congenital malformations, of which six with cardiovascular malformations
Nora et al. (1974)	Retrospective cohort study	Teratogenic history obtained in 733 women	Two lithium exposed pregnancies and both children were born with Ebstein anomaly
Weinstein and Goldfield (1975)	Cohort study	Exposed = 143	Cardiovascular abnormalities found in 9.1% of cases of exposure to lithium in 1st trimester
Kallen and Tandberg (1983)	Registry-based study	Exposed = 59 Other drugs = 38 Disease matched non-exposed = 80 Controls = 110	Four children with heart defects after lithium exposure. No cases of Ebstein anomaly
Jacobson et al. (1992)	Prospective cohort study	Exposed = 138 Controls = 148	No difference in the rate of major malformations
Reis and Kallen (2008)	Registry-based study	Exposed = 79	Eight children with congenital malformations, of which four with cardiac malformations
Diav-citrin et al. (2014)	Prospective cohort study	Exposed = 183 Disease matched non-exposed = 72 Controls = 748	Single center comparison: no difference in major malformations, increased risk of cardiovascular malformations (RR 7.23, 95% CI 1.97–26.53), not after excluding cases that spontaneously resolved (RR 5.78, 95% CI 0.82–40.65)
Patorno et al. (2017)	Registry-based study	Exposed = 663 Lamotrigine = 1945 Controls = 1,322,955	Increased risk of cardiac malformations after first trimester lithium exposure compared to controls (RR 1.65, 95% CI 1.02–2.68) and lamotrigine-exposed (RR 2.25, 95% CI 1.17–4.34)
Munk-Olsen et al. (2018)	Meta-analysis (six study sites)	Exposed = 727 Disease matched controls = 21,397	First trimester lithium exposure was statistically significant associated with congenital malformations (OR 1.62, 95% CI 1.12–2.33) but not with cardiac malformations in specific (OR 1.54, 95% CI 0.64–3.70)

*RR* risk ratio, *OR* odds ratio, *CI* confidence interval

In multiple investigations, lithium treatment during pregnancy has been associated with cardiovascular malformations, including Ebstein anomaly (Weinstein and Goldfield 1975; Schou et al. 1973; Nora et al. 1974; Patorno et al. 2017). Ebstein anomaly is a congenital malformation characterised by an abnormal development of the tricuspid valve and the right ventricle, with highly variable prognosis. The prevalence in the normal population is estimated to be about 1 per 20,000 live births (Lupo et al. 2011). The association with lithium use during pregnancy was first reported in the 1970s investigation on the Register of Lithium Babies (Weinstein and Goldfield 1975; Schou et al. 1973). Based on the data from the Register of Lithium Babies, Nora et al. estimated a fivefold increase in the risk of congenital heart-disease and about a 400-fold increase in the risk of Ebstein anomaly (Nora et al. 1974). In contrast, case control studies in children born with Ebstein anomaly or other cardiovascular malformations did not find an association with lithium exposure (Zalzstein et al. 1990; Boyle et al. 2016; McKnight et al. 2012; Correa-Villasenor et al. 1994; Sipek 1989; Lisi et al. 2010). For a comprehensive summary of case–control studies we refer to a review and meta-analysis by McKnight et al. (2012). A registry based case control study of 264 Ebstein anomaly cases by Boyle et al. found an association with maternal mental health problems in general but not with lithium use (Boyle et al. 2016).

Two studies on congenital malformations in general have yielded contradicting results, with one study reporting a high rate of congenital malformations after in utero exposure to lithium (Reis and Kallen 2008) and another study reporting no association between lithium exposure and congenital malformations (Jacobson et al. 1992). Additionally, several case reports have been published on congenital diaphragmatic hernia (Hosseini et al. 2010), goiter (Frassetto et al. 2002; Nars and Girard 1977), cardiovascular complications (Park et al. 1980; Long and Willis 1984; Arnon et al. 1981; Wilson et al. 1983), bilateral hip dislocation (Deiana et al. 2014) and neural-tube defect (Jacobson et al. 1992). In general, sample sizes of these clinical investigations are considered too small to study rare congenital malformations.

Recently, three cohort studies with large sample sizes, have provided more evidence on the matter (Munk-Olsen et al. 2018; Patorno et al. 2017; Diav-Citrin et al. 2014). Diav-Citrin et al. compared the rate of congenital abnormalities in lithium exposed pregnancies, disease matched and nonteratogenic-exposed pregnancies (Diav-Citrin et al. 2014). The occurrence of cardiovascular anomalies was higher in the lithium-exposed group although this difference was not significant after excluding the anomalies that resolved spontaneously. Patorno et al. used register data from Medicaid in the U.S. to study 1,325,563 pregnancies of which 663 were exposed to lithium and 1945 exposed to lamotrigine (Patorno et al. 2017). They found a dose dependent association between lithium exposure and cardiac malformations, including Ebstein anomaly. The adjusted risk ratio for cardiac malformations was calculated to be 1.65 compared to controls and 2.25 compared to lamotrigine-exposed. The risk of cardiac malformations was estimated to be in the order of one additional case per 100 live births. The same study found no association between lithium exposure and noncardiac malformations. In contrast, in a shared-protocol meta-analysis of six study sites the risk of major malformations (including cardiac malformations) was increased in lithium-exposed pregnancies (OR 1.62, 95% CI 1.12–2.33) compared to non-exposed pregnancies in mothers with a mood disorder, while there was no statistically significant increase in the risk of cardiac malformations (Munk-Olsen et al. 2018).

While this evidence is not conclusive it is recommended that it is discussed with women who seek advice on treatment of bipolar disorder either pre-pregnancy or during pregnancy. One option would be to taper lithium during the first trimester although the risk of relapse needs to weighed if considering this option. In the case of lithium continuation, fetal anomaly ultrasound including detailed fetal cardiac scanning, should be offered at 20 weeks gestational age. This could also be advised at 16 weeks (Galbally et al. 2010). In the case of detection of a cardiac malformation, information, guidance and counselling can be offered as early as possible. Although the pathophysiology of the association between lithium and congenital malformations is unclear, it might be related to lithium’s inhibition of the glycogen synthase kinase-3β (GSK3β) (Young 2009). GSK3β expression is of importance for the Wnt signaling pathway, which is of influence on cardiac and vascular development in the embryo (Corada et al. 2010; Jope 2003).

### Neonatal outcomes

Two studies found an increased risk of preterm birth in women with lithium use during pregnancy when compared to controls (Diav-Citrin et al. 2014; Troyer et al. 1993). In contrast, three studies including the meta-analysis of six studies reported no difference in the rate of preterm birth between lithium exposed pregnancies and controls (Newport et al. 2005; Munk-Olsen et al. 2018; Jacobson et al. 1992). In addition, most studies do not find differences in birth weight except for one small study in which lithium-exposed neonates had a higher birth weight (Newport et al. 2005; Munk-Olsen et al. 2018; Jacobson et al. 1992; Diav-Citrin et al. 2014; Troyer et al. 1993).

Lithium exposure is associated with increased risk of neonatal complications. Newport et al. found an association between high infant lithium concentrations and lower 1-min Apgar scores, higher rate of central nervous system and neuromuscular complications and longer duration of hospital stays (Newport et al. 2005). In a cohort of 19 babies exposed to lithium during pregnancy, 8 were admitted to a special care unit post-delivery (Frayne et al. 2017). This high rate of neonatal admissions was confirmed in a large meta-analysis of six study sites (Munk-Olsen et al. 2018). Additionally, there are case reports on neonatal lithium toxicity (Kozma 2005; Flaherty and Krenzelok 1997; Morrell et al. 1983; Woody et al. 1971; Wilbanks et al. 1970; Stothers et al. 1973), nephrogenic diabetes insipidus (Pinelli et al. 2002), and jaundice (Connoley and Menahem 1990). In a review of case reports, Kozma further reports respiratory problems, hypotonia, lethargy, poor drinking ability, thyroid problems, cyanosis, hypoglycemia and polyuria (Kozma 2005). Normal neonatal outcome was reported in the study from Silverman et al. (1971).

Because of potential problems in the neonatal period after in utero exposure to lithium, we recommend that delivery should take place in a specialised hospital with advanced neonatal care available immediately after delivery. In Table 3 we present the results of studies on neonatal outcome.

**Table 3:** Neonatal outcome after lithium treatment during pregnancy: findings from clinical cohort studies

Study	Design	Sample size	Findings
Jacobson et al. (1992)	Prospective cohort study	Exposed = 138 Controls = 148	No difference in the rate of preterm birth Higher birthweight in lithium exposed neonates
Troyer et al. (1993)	Cohort study	Exposed = 60 Disease matched non-exposed = 290	Cohort of manic-depressive women: risk ratio for prematurity of 2.54 No difference in birthweight
Newport et al. (2005a, b)	Cohort study	Exposed = 24	Lower Apgar scores, longer hospital stays and higher rates of CNS and neuromuscular complications in infants with high lithium levels No statistically significant association with preterm birth or low birth weight
Diav-citrin et al. (2014)	Prospective cohort study	Exposed = 183 Disease matched non-exposed = 72 Controls = 748	2.3 times higher rate of preterm delivery in exposed group (13.7% versus 6.0%) No differences in birth weight
Frayne et al. 2017	Cohort study	Exposed = 19	Eight neonates admitted to a special care unit
Munk-Olsen et al. (2018)	Meta-analysis (six study sites)	Exposed = 727 Disease matched controls = 21,397	No association between lithium exposure in utero and preterm birth (OR 1.24, 95% CI 0.83–1.84), low birth weight (OR 0.98, 95% CI 0.72–1.35) or small for gestational age (OR 0.90, 95% CI 0.67–1.21) A significant higher rate of neonatal admission (OR 1.62, 95% CI 1.12–2.33)

*OR* odds ratio, *CI* confidence interval

### Long term developmental outcome

It is assumed that the fetal environment influences lifetime disease risk based on Barker’s hypothesis of Developmental Origins of Health and Disease (DOHaD) (Barker 1990; Schlotz and Phillips 2009). This hypothesis proposes that exposure during fetal development can result in permanent physiological and metabolic changes, which modify disease risk through life. Prenatal exposure to lithium may therefore have consequences on development and health outcomes well beyond infancy. Indeed, results from preclinical studies in mice, rats and zebrafish show neurodevelopmental deficits (Poels et al. 2018). Clinical data are scarce, four small clinical cohort studies have investigated long term neurodevelopmental outcomes. The results of these studies are presented in Table 4. Schou used data from the Scandinavian Register of Lithium Babies to compare the mothers’ subjective retrospective assessment of their children’s development in lithium-exposed children (n = 60) and their non-exposed siblings (n = 57) and found no difference (Schou 1976). In a prospective multicenter study, there was no difference in the age of attainment of major developmental milestones in lithium-exposed children compared to non-exposed children (Jacobson et al. 1992). Another study examined 15 lithium-exposed children at the age of 3–15 years old and used standard validated tests to assess growth, neurological, cognitive and behavioural outcomes (van der Lugt et al. 2012). Most children scored lower on the performance Block patterns when compared to the general population although this difference was not statistically significant. Growth and behavioural development was within normal range. One child in this study was diagnosed with minor neurological dysfunction without clinical implications. A recent study compared the intelligence quotient (IQ) in children with in utero exposure to lithium (n = 20), non-exposed children of mothers with a mood disorder (n = 8) and controls (n = 11) and reported no difference in total, performance or verbal IQ (Forsberg et al. 2017).

**Table 4:** Neurodevelopmental consequences of intrauterine exposure to lithium: findings from clinical cohort studies

Study	Design	Sample size	Follow-up	Findings
Schou (1976)	Prospective cohort study	Exposed = 60 Controls = 57	Mean = 7 years	No difference in development based on questionnaire filled out by the mother
Jacobson et al. (1992)	Prospective cohort study	Exposed = 22 Controls = n.r.	1–9 years, mean = 61 weeks	No difference in attainment of milestones
van der Lugt et al. (2012)	Cohort study	Exposed = 15	3–15 years	Normal developmental milestones (n = 15), minor neurological dysfunction (n = 1), low verbal + total IQ, normal performance IQ (n = 1), subclinical anxiety problems (n = 2), subclinical oppositional problems (n = 1)
Forsberg et al. (2017)	Cohort study	Exposed = 20 Disease matched non-exposed = 8 Controls = 11	4–5 years	No differences in total, performance and verbal IQ

*IQ* intelligence quotient, *n.r.* not reported

In summary, while preclinical evidence does point to possible developmental effects of perinatal exposure to lithium, this is not found in clinical investigations. Due to methodological weaknesses of the published clinical studies (e.g. small sample sizes, lack of control group and subjective outcome measures) no conclusion can be drawn from these results and more research is needed to provide an estimation of the risk for the developing child.

## Lithium use during pregnancy

- Maintenance of lithium during pregnancy is effective in the prevention of relapse during pregnancy and the postpartum period.
- The first and second trimester are characterized by a significant decrease in blood levels for lithium.
- Fetal anomaly ultrasound including detailed fetal cardiac scanning, should be offered at 20 weeks gestational age.
- In the third trimester, weekly monitoring of lithium blood levels is recommended. Preferably, lithium blood levels should be measured before and 24 h after delivery.
- Lithium blood level, TSH and free T4 should be evaluated in umbilical cord blood sample.
- Lithium use during pregnancy has not been associated with obstetric complications. However, the association with preterm birth and birthweight remains uncertain.
- Lithium exposure during the first trimester is associated with congenital malformations in several studies, recent studies estimate the risk lower than previously reported. Tapering of lithium during the first trimester should be considered but weighed against the risks of relapse.
- Lithium exposure is associated with increased risk of neonatal complications. Lithium-exposed neonates should be observed directly post-delivery.
- Little is known about the developmental consequences of intrauterine exposure to lithium.

## Lithium use postpartum

### Efficacy

Women with a history of bipolar disorder or postpartum psychosis are at extremely high risk of relapse postpartum. Few clinical studies have investigated the efficacy of pharmacotherapy when it is initiated immediately after delivery, as a prophylactic strategy in women who have not been treated during pregnancy. A meta-analysis showed that patients with bipolar disorder using prophylactic pharmacotherapy during the postpartum period had a lower relapse rate (N = 98; 29%, 95% CI 16–47) compared with those who remained medication free (N = 107; 65%, 95% CI 55–73) (Wesseloo et al. 2016). Off these 98 women, 38 started prophylactic treatment during pregnancy while 22 were medication free during pregnancy and initiated prophylaxis immediately postpartum. For the remaining women information regarding the timing was unavailable or they were on chronic maintenance treatment. Numbers are very small but in all studies on prophylactic treatment with lithium postpartum, women with bipolar disorder had significantly lower rates of postpartum relapse compared to medication free women (Bergink et al. 2012; Austin 1992; Cohen et al. 1995). In contrast, valproate failed to demonstrate significant prophylactic benefits (Wisner et al. 2004) and further investigation of second generation antipsychotics is warranted. In our previous work we have recommended distinct perinatal treatment algorithms for women with bipolar disorder and women with a history of psychosis limited to the postpartum period. In women with bipolar disorder, prophylaxis during pregnancy increases the likelihood of maintaining mood stability during pregnancy and preventing postpartum relapse. In women with a history of psychosis limited to the postpartum period, prophylactic treatment immediately after birth is appropriate (Bergink et al. 2016). In this group of women with a history of postpartum psychosis, the established efficacy of lithium makes it the drug of first choice for postpartum prophylaxis.

### Dosing and monitoring of blood level

Lithium prophylaxis has demonstrated efficacy in reducing postpartum episodes. However, the dosing and duration of prophylaxis is unknown. We recommend relapse prevention prophylaxis in women with bipolar disorder with a higher lithium target level (for example 0.8 mmol/L) during the first month postpartum. Given that the relapse risk is high particularly in the first month postpartum, we follow the view that the benefits of higher lithium target blood levels in the first month postpartum outweighs the potential risks. We recommend to start lithium on the first evening after delivery and with a dose to target blood level of 0.8–1.0 mmol/L to optimize relapse prevention. In our previous work, we observed that normalization of renal function can take up to a few weeks after delivery as both mean lithium and creatinine blood levels were higher in the postpartum period than in the preconception period (+ 9% and + 7% respectively) (Wesseloo et al. 2017). Therefore, we recommend twice weekly monitoring of lithium blood levels for the first 2 weeks postpartum. Women with bipolar disorder on maintenance treatment with lithium might want to change to their preconception dose and blood level after 1 month postpartum. For those women who want to taper their lithium dose (i.e. women with isolated postpartum psychosis/mania in history, or women with bipolar disorder without regular maintenance treatment) we advise to commence tapering after 3 months postpartum.

### Breastfeeding

Clinical guidelines generally discourage breastfeeding in women treated with lithium due to the possible risk of lithium toxicity in the newborn (National Collaborating Centre for Mental H 2014). Furthermore, the lack of continued sleep during puerperium might also increase the risk of maternal relapse. Lithium is excreted into breast milk and the elimination rate in infants is lower than in adults, which may cause higher exposure levels in infants. However, there is a lack of data from clinical investigations on this topic. In Table 5 we present the results of clinical studies on infant lithium exposure through lactation. Some case studies have estimated serum lithium levels to be about one-half of maternal serum lithium levels (Frew 2015; Schou and Amdisen 1973) while others estimated levels closer to one quarter of the mothers’ levels (Bogen et al. 2012; Sykes et al. 1976). The larger study available for lithium and breastfeeding consists of 11 mother infant pairs with a calculated infant lithium dose as 0–30% of the maternal dose per kilogram bodyweight, based on the daily milk intake and lithium levels measured in breast milk (Moretti et al. 2003). Unfortunately, serum lithium levels were only available in two mother–infant pairs. In one pair lithium serum levels of the infant achieved 17–20% of the maternal serum level while in the other infant this was calculated to be 50%. No adverse effects were observed in the lithium exposed infants. Viguera et al. measured lithium levels in breast milk, maternal serum and infant serum in ten mother child pairs from eight to 27 weeks postpartum (Viguera et al. 2007). Based on these measurements it was estimated that infant lithium levels in serum were about one quarter of the lithium levels in serum of the mother. This estimation was lower than previous reports (Frew 2015; Schou and Amdisen 1973; Moretti et al. 2003). Lithium exposure through breastmilk was generally well tolerated by the infants in this study although one infant developed elevated levels of TSH which, normalised after the mother discontinued lithium treatment. Three other infants showed transient elevations in blood urea nitrogen and creatinine levels. In summary, there is a lack of sufficient information on infant lithium levels and the consequences of lithium exposure through breast milk. Due to the lack of information and the possible nephrotoxic effects of lithium in infants, in combination with the vulnerability of the developing neonatal kidneys and the risk of dehydration associated with the neonatal period, breastfeeding while on lithium treatment is discouraged in many national guidelines and individual centers worldwide (Galbally et al. 2018).

**Table 5:** Summary of the results from clinical studies on infant lithium exposure through lactation

Study	Design	Sample size	Findings
Schou et al. (1973)	Case series	8 mother–infant pairs	Infant/maternal serum lithium concentration of 1/2 in first week and 1/3 during the following weeks
Sykes et al. (1976)	Case report	1 mother–infant pair	Breast milk lithium level of 1/4 of maternal serum level, infant had good excretion of lithium into urine
Moretti et al. (2003)	Case series	11 mother–infant pairs	Infant lithium dose of 0–30% of the maternal dose/kg Infant serum level of 17–50% of maternal serum level
Viguera et al. (2007a, b)	Case series	10 mother–infant pairs	Mean infant serum level of 0.16 meq/L (range 0.09–0.25) In four infants: transient elevations of TSH, blood urea nitrogen and creatinine
Bogen et al. (2012)	Case series	3 mothers with 4 infants	Infant lithium levels ranged from 10 to 17% of maternal levels at 1 month postpartum
Frew (2015)	Case report	1 mother–infant pair	Infant/maternal serum lithium concentration ratio of 0.58. No adverse events

## Lithium use postpartum

- When lithium is discontinued during pregnancy, lithium should be restarted immediately after delivery and is an effective strategy for relapse prevention in the immediate postpartum.
- For women with an isolated episode of postpartum psychosis or mania in history lithium prophylaxis immediately after delivery is effective for relapse prevention, there is no need to use lithium during pregnancy.
- Consider a high target therapeutic lithium level immediately after delivery and during the first month postpartum to optimize relapse prevention (e.g., 0.8–1.0 mmol/L).
- Obtain lithium blood levels twice weekly during the first 2 weeks postpartum.
- Breastfeeding while taking lithium is not recommended.

## Summary and discussion

The aim of this review was to provide a broad range of information and clinical guidance regarding lithium use during pregnancy and the postpartum period. Since it was our aim to give a broad overview of the literature from a clinical perspective we opted for a narrative review rather than a systematic review or meta-analysis. The clinical recommendations in this review article are suggestions based on the available scientific information and clinical experience of the authors. Readers should note that recommendations were not formulated within the context of a guideline procedure.

Women of childbearing age requiring mood stabilisation should be given the opportunity to weigh the risks and benefits of lithium treatment during pregnancy and the postpartum period, and to develop an individualised treatment plan together with their healthcare providers in a specialised centre (Bergink and Kushner 2014; Yonkers et al. 2004). Antenatal care should take place in a multidisciplinary setting, with close collaboration between psychiatric and obstetric services. During pregnancy and the postpartum period women with bipolar disorder should be closely monitored. The possible risks for the unborn child, such as the risk of congenital malformations need to be carefully weighed against the risk of maternal relapse. The pros and cons of discontinuation of medication need to be compared with the pros and cons of continuing medication. In this context it is important to note that also relapse of bipolar disorder carries a fetal risk. High maternal stress but also high-risk behaviour, such as alcohol or drug use or lack of compliance to antenatal care are associated with adverse fetal outcomes.

Switching to maintenance therapy with lamotrigine before conception should be considered as the efficacy of lamotrigine in prevention of postpartum relapse is not inferior to lithium (Wesseloo et al. 2017) and there are no risks of congenital malformations associated with its use (Patorno et al. 2017). However, the efficacy of lamotrigine in the prevention of postpartum episodes was established in a group of women with a high vulnerability to depressive episodes and lamotrigine is not effective in the prevention of mania. Moreover, the efficacy of lamotrigine in the prevention of relapse during pregnancy is not yet investigated. Maintenance therapy with second generation antipsychotics is an alternate treatment option (National Collaborating Centre for Mental H 2014). Importantly, the use of second generation antipsychotics during pregnancy is not associated with an increased risk of congenital malformations (Petersen et al. 2016; Huybrechts et al. 2016). However, a recent Medicaid study found an increased risk of gestational diabetes associated with the continuation of quetiapine and olanzapine during pregnancy (Park et al. 2018) and there is uncertainty on the long-term impact on neurodevelopment (Poels et al. 2018). Notably, the efficacy of second generation antipsychotics in relapse prevention during the perinatal period is not yet properly investigated in women with bipolar disorder. Moreover, antipsychotics are known to be less effective than lithium in maintenance treatment for bipolar disorder outside the perinatal period (Geddes and Miklowitz 2013).

When providing advice to the individual patient, knowledge about past treatment efficacy should also be taken into account. In women with a history of severe bipolar episodes and a good effect on lithium therapy, continuation of lithium might be preferred in order to prevent relapse. When lithium therapy is continued during pregnancy, regular antenatal visits are warranted for checking lithium blood levels, evaluation of fetal growth, and for monitoring signs of preterm labour. Detailed fetal anomaly scanning, including detailed fetal cardiac scanning should be offered at 20 weeks gestational age or maybe earlier in the future. Furthermore, evaluation of maternal thyroid (TSH and free T4) levels and kidney function is recommended (Trimbos-instituut 2015). Delivery should take place in a specialised hospital where psychiatric and obstetric care for the mother is provided and neonatal evaluation and monitoring of the child can take place immediately after birth.

More investigations are required on the development of children exposed to lithium in utero as the studies that have been published so far provide insufficient information to properly advise women.

We recommend against breastfeeding while on lithium treatment given both the paucity and the poor quality of the available clinical reports. However, we are aware of the differences in clinical recommendations between guidelines and authors. For instance, a recent systematic review by Pacchiarotti et al. studied the same literature as reported in this review paper but they concluded that lithium levels in the infant were low and breastfeeding should be permitted through an individualized approach (Pacchiarotti et al. 2016). Clinicians that do recommend breastfeeding should publish their findings with comprehensive data on lithium levels in serum and breast milk, as well as infant outcomes including neurological, renal and thyroid function. In this way more knowledge will be available in order to develop evidence based recommendations.
